# Supplementary material for: Bayesian refinement of protein structures and ensembles against SAXS data using molecular dynamics
Source: PLoS Comput Biol. 2017 Oct 18;13(10):e1005800. doi: 10.1371/journal.pcbi.1005800 (PMC5662244; doi:10.1371/journal.pcbi.1005800)
Supplement: S1 Archive — (BZ2) [file pcbi.1005800.s011.tar.bz2 › gromacs_4.62_bayesian_saxs_refi/share/html/online/g_principal.html]

g\_principal


| g\_principal Main Table of Contents |

**VERSION 4.6  
Sat 19 Jan 2013**

---

### Description

g\_principal calculates the three principal axes of inertia for a group
of atoms.

### Files

| option | filename | type | description |
| --- | --- | --- | --- |
| **-f** | traj.xtc | Input | Trajectory: xtc trr trj gro g96 pdb cpt |
| **-s** | topol.tpr | Input | Structure+mass(db): tpr tpb tpa gro g96 pdb |
| **-n** | index.ndx | Input, Opt. | Index file |
| **-a1** | axis1.dat | Output | Generic data file |
| **-a2** | axis2.dat | Output | Generic data file |
| **-a3** | axis3.dat | Output | Generic data file |
| **-om** | moi.dat | Output | Generic data file |

### Other options

| option | type | default | description |
| --- | --- | --- | --- |
| **-[no]h** | bool | no | Print help info and quit || **-[no]version** | bool | no | Print version info and quit || **-nice** | int | 19 | Set the nicelevel || **-b** | time | 0 | First frame (ps) to read from trajectory || **-e** | time | 0 | Last frame (ps) to read from trajectory || **-dt** | time | 0 | Only use frame when t MOD dt = first time (ps) || **-tu** | enum | ps | Time unit: fs, ps, ns, us, ms or s || **-[no]w** | bool | no | View output .xvg, .xpm, .eps and .pdb files || **-[no]foo** | bool | no | Dummy option to avoid empty array |

---

http://www.gromacs.org  
gromacs@gromacs.org
